# Tofacitinib treatment for psoriatic skin lesions associated with Aicardi-Goutières syndrome 7/Singleton-Merten syndrome 1

**DOI:** 10.1186/s13023-025-03675-7

**Published:** 2025-04-02

**Authors:** Shanice Beerepoot, Lucas Grinwis, Adeline L. Vanderver, Marjo S. van der Knaap, Taco W. Kuijpers

**Affiliations:** 1 Department of Child Neurology, Amsterdam Leukodystrophy Center, Emma Children’S Hospital, Amsterdam University Medical Centers, VU University, 1081 HV Amsterdam, The Netherlands; 2 Amsterdam Neuroscience, Cellular & Molecular Mechanisms, 1081 HV Amsterdam, The Netherlands; 3 Center for Translational Immunology, University Medical Center Utrecht, 3584 CX Utrecht, The Netherlands; 4 Princess Máxima Center for Pediatric Oncology, 3584 CS Utrecht, The Netherlands; 5 Department of Pediatric Immunology, Rheumatology and Infectious Diseases, Amsterdam University Medical Centers, Emma Children’S Hospital, University of Amsterdam, Amsterdam, The Netherlands; 6 Amsterdam University Medical Centers, Department of Experimental Immunology, Amsterdam Institute for Infection and Immunity, University of Amsterdam, Amsterdam, The Netherlands; 7 Division of Neurology, The Children’S Hospital of Philadelphia, Philadelphia, PA USA; 8 Department of Neurology, Perelman School of Medicine, University of Philadelphia, Philadelphia, PA USA; 9 Department of Functional Genomics, Center for Neurogenomics and Cognitive Research, Vrije Universiteit Amsterdam, 1081 HV Amsterdam, The Netherlands; 10 Amsterdam University Medical Centers, Location AMC, Meibergdreef 9, 1105 AZ Amsterdam, The Netherlands

**Keywords:** Aicardi-Goutières syndrome 7, Interferonopathy, Janus kinase (JAK) inhibitor, Singleton-Merten syndrome 1, Tofacitinib

## Abstract

The purpose of this letter to the editor is to illustrate the effect of tofacitinib on psoriatic skin lesions in a patient with Aicardi–Goutières syndrome (AGS) type 7/Singleton-Merten syndrome 1. AGS is characterized by an encephalopathy of variable severity and systemic autoinflammatory manifestations due to continuous type I interferon (IFN) induction. While traditional JAK 1/2 inhibitors like baricitinib and ruxolitinib have proven effectiveness for systemic inflammatory symptoms, they face reimbursement issues in some countries. Tofacitinib, a JAK 1/3 inhibitor, significantly improved psoriatic skin lesions in our patient without the need for additional immunosuppressive therapy. Within one month of starting tofacitinib, psoriatic rashes and ulcerative skin lesions markedly improved, in the absence of a reduction in the IFN-stimulated gene signature or CD169 expression on monocytes. The clinical benefits persisted until the treatment was discontinued, after which symptoms recurred. Resuming tofacitinib treatment again led to improvement. No adverse effects were observed. This case highlights the potential of tofacitinib as a clinically effective treatment for psoriatic skin lesions in AGS and offers a viable alternative for JAK 1/2 inhibitors for this target symptom. Further studies are needed to confirm the long-term safety of JAK 1/3 inhibitors in AGS as well as their possible efficacy and dosing to address other systemic symptoms or neurologic manifestations.

## Dear editor,

Aicardi–Goutières syndrome (AGS) is a monogenic type-I interferonopathy characterized by an encephalopathy and autoinflammatory systemic manifestations. Patients may suffer from variably severe neurological disability, recurrent sterile fevers, glaucoma, chilblains, organ dysfunction, arthritis, and skin abnormalities, including severe psoriasiform and ulcerative lesions [1].

Pathogenic variants in genes associated with AGS (*TREX1, RNASEH2B, RNASEH2C, RNASEH2A, SAMHD1, ADAR, IFIH1, LSM11, RNU7-1*, in corresponding order associated to AGS types 1–9) result in constitutive induction of type I interferon (IFN) and upregulation of IFN-stimulated genes. The IFN-stimulated gene signature (interferon signature) measures the mRNA expression of a selection of IFN-stimulated genes in peripheral blood and is suggested as a surrogate marker of disease activity in assessing treatment efficacy in AGS [2]. Janus kinase (JAK) 1/2 inhibitors, such as baricitinib and ruxolitinib, have shown to be effective in blocking IFN I activation, resulting in decreased interferon signature and improved autoinflammatory systemic manifestations in patients with AGS [3]. The ability of JAK 1/2 inhibitors to affect neurological manifestations is hampered by their limited capacity to penetrate the blood–brain barrier, requiring high treatment doses [4].

Despite being repurposed for the treatment of AGS [3, 5, 6], JAK 1/2 inhibitors currently lack approval for AGS from the European Medicines Agency’s (EMA) and US Food and Drug Administration’s (FDA), leading to issues with reimbursement in some countries. Tofacitinib, a JAK 1/3 inhibitor with less inhibition of JAK2 and used to treat rheumatoid arthritis, inflammatory bowel disease, as well as psoriasis [7], might be an alternative therapeutic agent to treat autoinflammatory systemic manifestations in patients with AGS. JAK1/3 inhibitors may affect a distinct set of cytokine and growth factor receptors, thereby influencing various immune cells, including T cells, which play a role in immune-mediated skin manifestations [8, 9]. Additionally, its ability to effectively traverse the blood–brain barrier suggests a potential supplementary impact on neurological manifestations [10].

In one patient with AGS type 7, adjunctive treatment with tofacitinib to corticosteroids and cyclophosphamide resulted in substantial improvement of psoriasis lesions, interstitial lung disease, and autoimmune glomerulonephritis [11]. Improvement of skin lesions was also found in a patient with AGS type 5 treated with a combination of tofacitinib, corticosteroids and methotrexate [12]. Reduction of skin lesions and sterile febrile episodes were observed in a patient with AGS type 1 treated with tofacitinib and prednisone, although an elevated interferon signature persisted [13].

Here, we describe our experience with a patient with AGS type 7/Singleton-Merten syndrome 1, whom we treated with tofacitinib monotherapy.

**Fig. 1:**
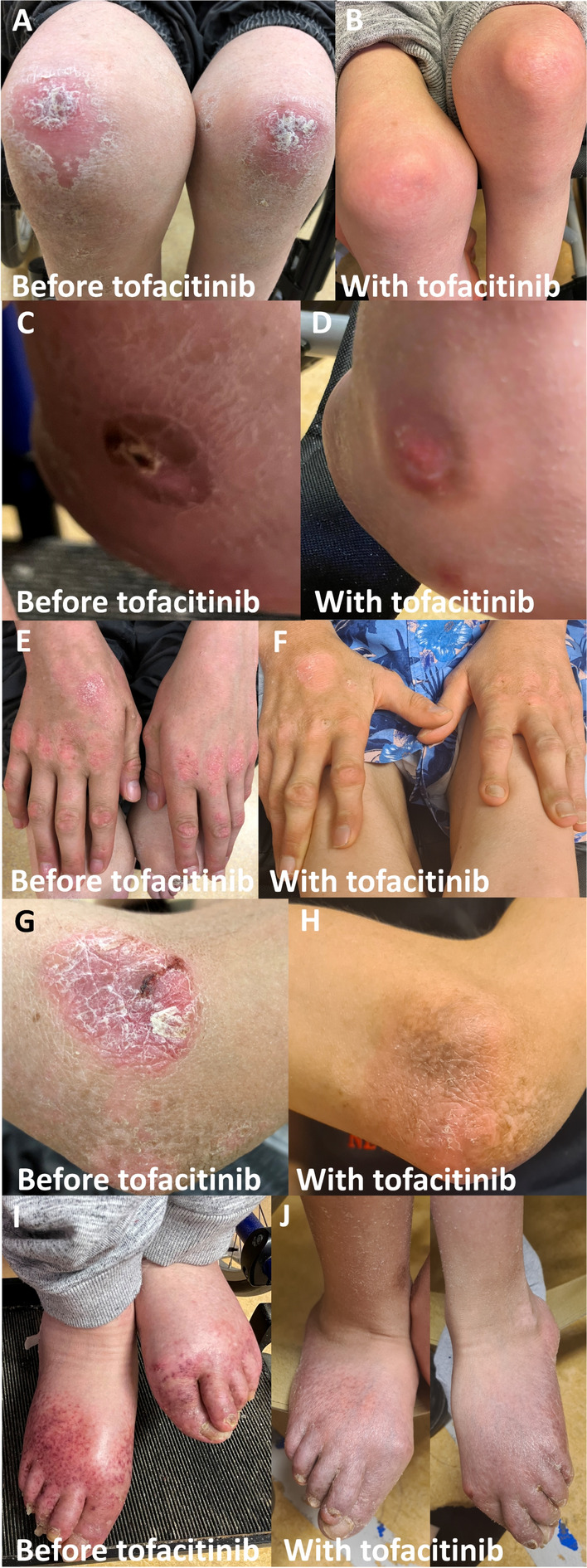
Psoriatic skin lesions of a patient with Aicardi-Goutières syndrome 7/Singleton-Merten syndrome 1 before and under treatment with tofacitinib. Psoriatic rash on the knees before treatment **A** and after 3 weeks treatment with tofacitinib **B**. Ulcerating skin lesion and swelling of the left ankle before treatment **C** and after 3 weeks treatment with tofacitinib **D**. Psoriatic rash on the hands and fingers before treatment **E** and after 3 weeks treatment with tofacitinib **F**. Psoriatic rash and ulcerating skin lesion on the left elbow before treatment **G** and 2 months after resumed treatment with tofacitinib **H**. Skin lesions and swelling of the feet before treatment **I** and 2 months after resumed treatment with tofacitinib **J**

**Fig. 2:**
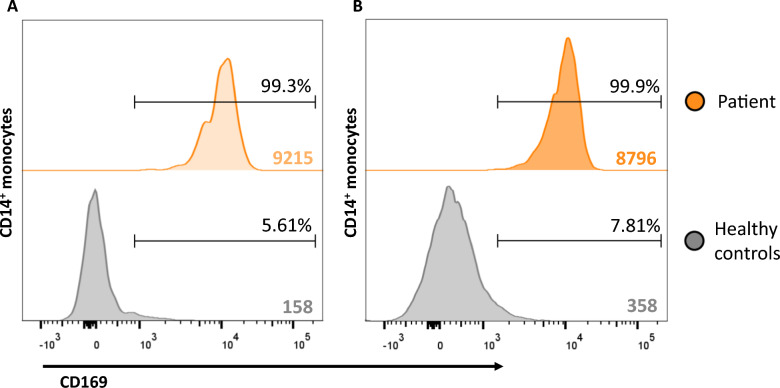
Surface expression of CD169 (SIGLEC1) on monocytes before (**A**) and under treatment with tofacitinib (**B**). Flow cytometry analysis shows stable surface expression of CD169 (SIGLEC1) on CD14 + monocytes before and under treatment with tofacitinib, evaluated by the percentage of cells with positive expression and the geometric mean fluorescence intensity (geoMFI) of the entire CD14 + monocyte population. The percentage of CD14 + monocytes expressing CD169 was 99.3% before treatment and 99.9% under treatment, with geoMFI values of 9215 and 8796, respectively. Two independent control samples are included for reference

The currently 22-year-old patient presented at 14 months with an episode of fever followed by motor regression. The patient developed a severe spastic paresis of the legs and cognitive impairment. Neuroimaging showed signal abnormalities in the periventricular and deep cerebral white matter, and bilateral calcifications in the basal ganglia and dentate nuclei. A maternally inherited monoallelic missense variant in the *IFIH1* gene (NM_022168.4, c.2465G > A, p.Arg822Gln), known to be pathogenic in AGS type 7/Singleton-Merten syndrome 1 [14], was identified. The patient developed multiple disease manifestations, including short stature, osteoporosis, dental dysplasia, glaucoma, aortic valve calcification, autoimmune thyroiditis, mild arthritis of hands and feet, and severe psoriatic rashes with ulcerating skin lesions of elbows, hands, hips, knees and feet (Fig. 1A, C, E, G, I). Local tacrolimus therapy was not consistently applied and not effective. With the aim of treating the skin lesions and arthritis, we prescribed tofacitinib as an alternative therapeutic agent for baricitinib and ruxolitinib, which did not qualify for reimbursement. Tofacitinib is approved by the FDA and EMA for treatment of psoriatic arthritis. Oral tofacitinib was initiated at 5 mg twice a day. Within 1 month, psoriatic rashes, as well as the ulcerative skin lesions, had markedly improved (Fig. 1B, D, F) and swelling and tenderness of the feet was decreased. No effect on the proximal interphalangeal joints of the index fingers was observed. In contrast to the clinical improvement of the skin, the interferon signature, calculated by normalizing gene expression levels for five IFN-stimulated genes (*IFI44*, *IFI44L*, *IFIT1*, *IFIT3*, and *MXA*) to the housekeeping gene *ABL* and applying the formula = ∑((log (2^-(CT-Abl))-Mcontrol)/SDcontrol) [15], minimally reduced after treatment initiation (21.03 to 18.17, normal range limit < 9.4). The clinical improvements were sustained until the patient discontinued treatment three months later for personal reasons. Skin lesions reappeared within the subsequent four months. The patient resumed tofacitinib treatment at the same oral dose, again resulting in improvement (Fig. 1H, 1J). These improvements have persisted to date, seven months after the second treatment initiation without a clear change in interferon signature (14.72) and persistent expression of CD169 (SIGLEC1) on monocytes (Fig. 2), one of the key markers for interferon activity [15]. No neurological changes were observed and apart from mild gastrointestinal complaints, no adverse effects have occurred. It is unknown at this time if higher dosing would be needed to change interferon signatures or neurologic features. Alternatively, these findings may suggest that the observed clinical improvements arise from mechanisms beyond JAK1 inhibition, potentially involving additional inflammatory pathways, or tissue-specific effects. The reproducible and objectively observable improvement of skin lesions with tofacitinib initiation and re-initiation, along with the recurrence of the skin lesions upon tofacitinib discontinuation supports a causal relationship with the treatment rather than a placebo effect or another confounding factor

.

In conclusion, tofacitinib administration in a patient with AGS type 7/Singleton-Merten syndrome 1 was well tolerated and had striking beneficial effects on the psoriatic skin lesions achieved within less than one month. Further studies in larger number of patients are needed to confirm the therapeutic effects of tofacitinib as a therapeutic alternative in AGS for systemic symptoms including skin lesions.

## Data Availability

The data that support this article are available from the corresponding author upon reasonable request.

## Abbreviations

AGS: Aicardi–Goutières syndrome
EMA: European medicines agency’s
FDA: Food and drug administration
geoMFI: Geometric mean fluorescence intensity
IFN: Interferon
JAK: Janus kinase
